# The Relations between Acute and Chronic Diseases

**Published:** 1902-12-13

**Authors:** 


### THE RELATIONS BETWEEN ACUTE AND CHRONIC DISEASES.

In addressing the Midland Medical Society Dr. Mitchell Bruce recently drew attention to a very important point in regard to the nature of the diseases from which many people are said to suffer, namely, that what we commonly call "acute disease" is in many instances nothing else than an acute illness in the course of a chronic disease. This is an aspect of the case which is often overlooked in practice, the acute element alone being recognised, while the chronic element—the disease proper, possibly one of great gravity—is ignored. As instances, Dr. Mitchell Bruce mentions the sudden supervention of acute obstruction in the course of a chronic intestinal growth which has gradually developed without a single symptom; the occurrence of hæmoptysis in a patient presenting no other symptom of tuberculosis, but who, for all that, turns out to be affected with that disease; and the occurrence of acute abdominal symptoms in patients suffering from chronic uterine or other pelvic disease.

But cases may occur in which the acute element may take the form of a substantive disease, and in such cases one may readily forget the underlying chronic element. For example, is not acute pleurisy with effusion an "acute disease"? Yet, on apparent recovery, the patient may drift into consumption, and a more careful inquiry into his past history may show reason for believing that he had been tuberculous all along. Acute pleurisy, indeed, would appear to be the disease of all others which is most likely to lead us astray; and not in relation to tuberculosis alone, for besides being the first symptom which attracts attention in certain grave diseases, there is frequently an association between acute affections of the pleura and lungs with chronic diseases within the abdomen, such as carcinoma and hydatid of the liver, gastric ulcer, and appendicitis.

But even when we have determined the existence of a chronic pathological process beneath an acute illness, the considerations which the discovery suggests are not always appreciated in their full practical bearings. An elderly patient, say, is in bed with severe bronchial catarrh. We recognise that she is thoroughly gouty. But now that she is suffering from an acute disease, do we apply that knowledge? Do we see the application of it? Do we deny her the champagne for which she craves to relieve the distress of the acute illness from which she is suffering? The influence of the discovery of an underlying chronic illness upon the prognosis and often upon the treatment of an acute attack is sufficiently obvious, and may be well illustrated by what happens often enough in kidney disease. The sudden appearance of albuminuria with general anasarca and other symptoms may often be traced to chronic nephritis with a fresh renal congestion instead of to the acute nephritis which the symptoms at first sight seem to suggest. But the connection is readily missed. Yet how different an aspect does it not give both to prognosis and to treatment, for in this case, as in that of a fresh deposit in an old case of quiescent and may be forgotten tuberculosis, however favourable the outcome of the acute illness may be, the chronic substratum will remain a source of possible mischief in the future.

It is only by thus recognising the relations between acute and chronic diseases that we can understand the true nature of many of the former. Acute illness when looked at by itself is put down to chill, exhaustion, injury, and other "causes" which, so far from being the real origin of the disease are but exciting circumstances which without the underlying cause would have been harmless. We must then recognise that certain so-called "acute diseases" are but phases of a chronic and abiding condition. For example, hæmoptysis, pleurisy, fistula in ano, certain varieties of peritonitis, glandular enlargements, and even the occurrence of simple continued fever with no discoverable location, are often but phases of a tuberculous process, of the existence of which they should serve as danger signals. And so with many other conditions.

